# Integrative Computational Modeling of Distinct Binding Mechanisms for Broadly Neutralizing Antibodies Targeting SARS-CoV-2 Spike Omicron Variants: Balance of Evolutionary and Dynamic Adaptability in Shaping Molecular Determinants of Immune Escape

**DOI:** 10.3390/v17060741

**Published:** 2025-05-22

**Authors:** Mohammed Alshahrani, Vedant Parikh, Brandon Foley, Gennady Verkhivker

**Affiliations:** 1 Keck Center for Science and Engineering, Graduate Program in Computational and Data Sciences, Schmid College of Science and Technology, Chapman University, Orange, CA 92866, USA; alshahrani@chapman.edu (M.A.); vedpar31@gmail.com (V.P.); brfoley@chapaman.edu (B.F.); 2 Department of Biomedical and Pharmaceutical Sciences, Chapman University School of Pharmacy, Irvine, CA 92618, USA

**Keywords:** SARS-CoV-2 spike protein, Omicron variants, antibody binding, immune escape, molecular dynamics, protein stability, mutational scanning, binding energetics, evolutionary mechanisms

## Abstract

In this study, we conducted a comprehensive analysis of the interactions between the receptor-binding domain (RBD) of the SARS-CoV-2 spike protein and four neutralizing antibodies—S309, S304, CYFN1006, and VIR-7229. Using integrative computational modeling that combined all-atom molecular dynamics (MD) simulations, mutational scanning, and MM-GBSA binding free energy calculations, we elucidated the structural, energetic, and dynamic determinants of antibody binding. Our findings reveal distinct dynamic binding mechanisms and evolutionary adaptation driving the broad neutralization effect of these antibodies. We show that S309 targets conserved residues near the ACE2 interface, leveraging synergistic van der Waals and electrostatic interactions, while S304 focuses on fewer but sensitive residues, making it more susceptible to escape mutations. The analysis of CYFN-1006.1 and CYFN-1006.2 antibody binding highlights broad epitope coverage with critical anchors at T345, K440, and T346, enhancing its efficacy against variants carrying the K356T mutation, which caused escape from S309 binding. Our analysis of broadly potent VIR-7229 antibody binding to XBB.1.5 and EG.5 Omicron variants emphasized a large and structurally complex epitope, demonstrating certain adaptability and compensatory effects to F456L and L455S mutations. Mutational profiling identified key residues crucial for antibody binding, including T345, P337, and R346 for S309 as well as T385 and K386 for S304, underscoring their roles as evolutionary “weak spots” that balance viral fitness and immune evasion. The results of the energetic analysis demonstrate a good agreement between the predicted binding hotspots, reveal distinct energetic mechanisms of binding, and highlight the importance of targeting conserved residues and diverse epitopes to counteract viral resistance.

## 1. Introduction

The emergence of the severe acute respiratory syndrome coronavirus 2 (SARS-CoV-2) has spurred extensive research into understanding its structure, infection mechanisms, and immune responses. The SARS-CoV-2 Spike (S) glycoprotein is central to viral transmission and immune evasion, characterized by remarkable conformational flexibility [1,2,3,4,5,6,7,8,9,10,11,12,13,14,15]. The S1 subunit of S protein includes the N-terminal domain (NTD), receptor-binding domain (RBD), and conserved subdomains SD1 and SD2. The NTD facilitates initial host cell attachment, while the RBD binds to the angiotensin-converting enzyme 2 (ACE2) receptor, transitioning between “up” and “down” conformations to modulate receptor and antibody accessibility [1,2,3,4,5,6,7,8,9,10,11,12,13,14,15]. SD1 and SD2 stabilize the prefusion state and orchestrate membrane fusion, highlighting the S protein’s adaptability and complexity [10,11,12,13,14,15,16,17,18]. Biophysical studies have revealed the thermodynamic and kinetic principles governing its functional transitions, emphasizing mechanisms that balance receptor binding, membrane fusion, and immune escape [16,17,18]. The extensive array of cryo-electron microscopy (cryo-EM) and X-ray structures of S protein variants of concern (VOCs) in various functional states, along with their interactions with antibodies, has provided significant insights into the virus’s adaptability [19,20,21,22,23,24,25].

A critical component of the immune response to SARS-CoV-2 is the production of antibodies that target various regions of the S protein, which plays a central role in viral entry into host cells. High-throughput yeast display screening and deep mutational scanning (DMS) have revolutionized our understanding of the escape mutation profiles associated with the RBD residues of the SARS-CoV-2 spike protein. These advanced techniques have enabled the mapping of the functional epitopes targeted by human anti-RBD neutralizing antibodies, leading to a comprehensive classification of these antibodies into distinct epitope groups (A–F) [26]. A recent study expanded this approach to characterize the epitope distribution of antibodies elicited by post-vaccination BA.1 infection and identified the mutational escape profiles for 1640 RBD-binding antibodies, which were classified into 12 epitope groups [27]. Notably, groups E and F correspond to class 3 and class 4 antibodies in earlier classification systems [28], highlighting their role in recognizing non-overlapping epitopes that contribute to a diverse immune response.

**Figure 1:**
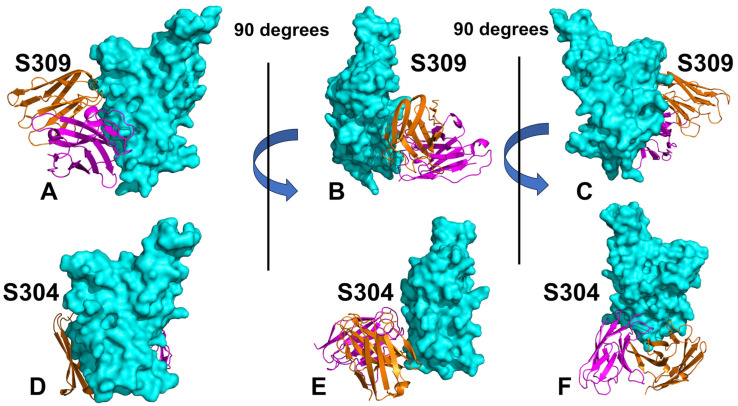
Structural organization of the SARS-CoV-2 S-RBD complexes with S309 (**A**–**C**) and S304 antibodies (**D**–**F**). The S-RBD structure is shown in the cyan surface. The heavy and light chains of antibodies are shown in orange and magenta-colored ribbons. The binding epitope residues are shown in the red surface. The structure of the S309 antibody in the complex with BA.1 RBD (pdb id 7YAD) is shown in the front view (**A**), side view (**B**), and back view (**C**). The structure of the S304 antibody with Wu-WT, RBD (pdb id 8WPY) is shown in the front view (**D**), side view (**E**), and back view (**F**).

S309 (group E) and S304 antibodies (group F1) represent two distinct classes of neutralizing antibodies (Figure 1A–C). Despite their shared ability to neutralize the virus, these antibodies exhibit significant differences in their binding epitopes, mechanisms of action, and neutralization profiles. S309 targets a conserved epitope near the N343 glycosylation site within the RBD (Figure 1A–C, Supporting Information, Figure S1A). This region is adjacent to but does not overlap with the ACE2-binding motif [29,30]. S309 does not directly compete with ACE2 for binding but rather induces conformational changes in the RBD that stabilize the “down” conformation, reducing the availability of the “up” state required for ACE2 engagement [29,30]. The epitope recognized by S309 is highly conserved across SARS-CoV-2 variants (Figure 1A–C) and related sarbecoviruses, such as SARS-CoV-1, making it effective against a broad range of coronaviruses [31]. S309 induces subtle conformational changes in the RBD that disrupt its interaction with ACE2. The cryo-EM structures of the BQ.1.1, XBB.1, and BN.1 RBDs bound to the S309 antibody and human ACE2 explain the preservation of antibody binding through conformational selection, altered ACE2 recognition, and immune evasion [32]. The structures demonstrated that S309 binds to both BQ.1.1 and XBB.1 RBDs [32] in a binding pose indistinguishable from that observed when it is bound to the Wu-WT [29,30] or the BA.1 RBD variant [33]. S309 binds near the ACE2-binding interface and forms extensive contacts with conserved residues, including T345, P337, L441, N343, A344, K356, and R346 (Supporting Information, Figure S1A). Together with residue N440, position R346 is a part of the epitope of S309 and important for binding S309 (Figure 1A–C, Supporting Information, Figure S1A). R346S alone was not sufficient to alter S309 binding, but R346S in combination with P337L enhanced resistance to S309 [34]. R346K in VOC Omicron BA.1.1 reduced sensitivity to S309 [35]. S309 can synergize with other antibodies targeting distinct epitopes, such as those within the ACE2-binding motif, to create a multi-layered defense against viral escape mutations [36]. Due to its conserved epitope and non-competitive mechanism, S309 demonstrates robust neutralization activity against variants of concern (VOCs), including Alpha, Beta, Gamma, Delta, and Omicron [37,38].

S304 binds to a unique epitope located near residues F377, Y380, and T385 within the RBD (Figure 1D–F). This region is also outside the ACE2-binding motif but differs significantly from the S309 epitope [39]. S304 sterically interferes with the spike protein’s ability to adopt the “up” conformation necessary for ACE2 binding (Figure 1D–F, Supporting Information, Figure S1B) and achieves this through steric hindrance rather than direct competition or allosteric modulation [39]. The structures of the ternary Omicron RBD-hACE2-S304 complex and the hACE2-bound Omicron spike with three reverse mutations (L371S, P373S, and F375S) by cryo-EM showed that S304 recognizes the inner face of the RBD buried in the down RBD configuration, and its binding to the spike is conformation-dependent [40]. In contrast to S309, S304 targets a structurally distinct epitope located on the inner face of the RBD β-sheet, involving residues Y369, N370, F374, S375, F377, K378, C379, T385, K386, S383, and Y380 (Supporting Information, Figure S1B).

The two recently discovered antibodies CYFN1006-1 and CYFN1006-2 (Supporting Information, Figures S1C and S2A,B) demonstrated consistent neutralization of all tested SARS-CoV-2 variants, outperforming SA55 [41]. These antibodies have binding epitopes overlapping with LY-CoV1404, REGN10987, and S309, located on the outer surface of the RBD (Supporting Information, Figure S2A,B). CYFN1006-1 binds to the outer side of the RBD (Supporting Information, Figures S1C and S2A,B) [41]. A yeast-display system combined with a machine learning (ML)-guided approach for library design enabled an investigation of a larger number of antibody variants and the identification of a class 1 human antibody designated as VIR-7229, which targets the receptor-binding motif (RBM), potently neutralizing SARS-CoV-2 variants, including EG.5, BA.2.86, and JN.1 [42]. The structures of VIR-7229-bound to XBB.1.5 and EG.5 variants showed that VIR-7229 binding induces conformational changes in the RBD, particularly in residues 473–489, and can accommodate both F456 and L456 in the corresponding genetic backgrounds [42], displaying an extraordinary tolerance to the epitope variability, with a high barrier for the emergence of resistance [43]. VIR-7229 binds to a structurally complex epitope within the RBM of the RBD, comprising 25 amino acid residues distributed across multiple regions of the RBD (Supporting Information, Figures S1D and S2C). These residues include 403, 405, and 409 located near the N-terminal edge of the RBM (Supporting Information, Figure S1D) [42].

Computer simulations have emerged as indispensable tools for elucidating the molecular mechanisms of the S protein, its interactions with the ACE2, and its ability to evade neutralizing antibodies at the atomic level [44,45,46,47,48]. These computational approaches provide unparalleled insights into the structural and energetic factors that govern viral–host interactions and immune escape strategies. By employing advanced techniques such as molecular dynamics (MD) simulations and Markov state models (MSMs), we have systematically mapped the conformational landscapes of Omicron subvariants like XBB.1 and XBB.1.5, as well as their complexes with ACE2 and antibodies [49]. Mutational scanning and binding analyses of XBB variants have identified key residues—including Y501, R498, Q493, L455F, and F456L—that contribute to epistatic interactions, strengthening ACE2 binding while conferring resistance to neutralizing antibodies [50,51]. Additionally, integration of AlphaFold2-based predictions with the ensemble-based analyses of the S protein-ACE2 complexes has enabled the identification of binding energy hotspots and epistatic networks involving critical residues like L455, F456, and Q493 in variants such as JN.1, KP.1, KP.2, and KP.3 [52]. Using MD simulations, mutational scanning of SARS-CoV-2 spike residues, and binding free energy computations, we examined mechanisms of broadly neutralizing antibodies: the E1 group (BD55-3152, BD55-3546, and BD5-5840) and F3 group (BD55-3372, BD55-4637, and BD55-5514) [53]. A comprehensive structural and energetic analysis of the RBD complexes with neutralizing antibodies from four distinct groups (A–D), including group A LY-CoV016; group B AZD8895 and REGN10933; group C LY-CoV555; and group D antibodies AZD1061, REGN10987, and LY-CoV1404, identified key binding hotspots and explored the evolutionary strategies employed by the virus to evade neutralization [54]. Previous studies also demonstrated that the S protein functions as an allosteric regulatory machine, leveraging its intrinsic flexibility to modulate receptor binding and immune evasion [55,56,57,58]. Electrostatic interactions have emerged as another critical thermodynamic force governing the binding of the S protein to ACE2 and its resistance to antibodies [59]. Several investigations suggested that electrostatic forces also play a crucial role in the interaction between the S protein and RBD, with the heparan sulfate of the extracellular matrix supporting the co-receptor mechanism that drives the interaction between RBD and ACE2 [60,61,62].

The evolution of SARS-CoV-2, particularly within the Omicron lineage, has been characterized by the emergence of subvariants with enhanced immune evasion and transmissibility, most recently the JN.1, KP.2, and KP.3 variants [63]. JN.1 has evolved into multiple subvariants, each with distinct mutations that contribute to immune evasion and transmissibility (Supporting Information, Table S1). KP.2 (R346T, F456L, V1104L) and KP.3 (R346T, L455S, F456L, Q493E, V1104L) share the F456L mutation, which contributes significantly to antibody escape and makes KP.3 the most immune-evasive and rapidly growing JN.1 sublineage [63]. The F456L mutation enhances the binding potential of Q493E, leading to stronger receptor interactions and providing an evolutionary advantage for incorporating additional immune-evasive mutation [64,65]. Many studies have suggested that functionally balanced substitutions that optimize tradeoffs between immune evasion, high ACE2 affinity, and sufficient conformational adaptability might be a common strategy of the virus evolution and serve as a primary driving force behind the emergence of new Omicron subvariants [66,67,68].

The cross-neutralization activity of antibodies against Omicron variants reflects a dynamic equilibrium shaped by multiple factors, including the energetic contributions of specific molecular interactions, the distribution of escape hotspots across the protein, and the selective pressures exerted by diverse antibody repertoires. In this study, we expand upon existing research by examining the molecular mechanisms of antibody binding through a comparative analysis of dynamics and energetics of S309, S304, GYFN-1006, and VIR-7229 antibody complexes with the RBD of the SARS-CoV-2 S protein. Using MD simulations, we examined dynamic ensembles that represent the structural variability and flexibility of these systems and allowed us to examine how mutations in the RBD influence the stability and adaptability of the binding interfaces. By analyzing the MD trajectories, we identified key regions of the RBD that exhibit significant conformational changes upon antibody binding. Functional motions were further dissected using principal component analysis (PCA), which revealed collective modes of motion that dominate the dynamics of the complexes. To systematically assess the impact of mutations on antibody binding, we conducted mutational scanning of RBD residues across the S-antibody complexes and generated mutational sensitivity heatmaps, which highlighted “escape hotspot” centers—regions where mutations significantly reduced antibody binding affinity. These hotspots often coincided with epitope residues directly contacted by the antibodies, but intriguingly, some non-contact residues also emerged as critical contributors due to their roles in maintaining the structural integrity of the binding interface. Mutational profiling and binding free energy analysis identified key residues crucial for antibody binding, underscoring their roles as evolutionary “weak spots” that balance viral fitness and immune evasion. The results of this study dissect the distinct energetic mechanisms of binding and importance of targeting conserved residues and diverse epitopes to counteract viral resistance. The results of this study underscore the diversity of binding mechanisms employed by different antibodies and the molecular basis for high affinity and excellent neutralization activity of the latest generation of antibodies.

## 2. Materials and Methods

### 2.1. Molecular Dynamics Simulations

The crystal and cryo-EM structures of the Omicron RBD complexes were obtained from the Protein Data Bank [69]. For simulated structures, hydrogen atoms and missing residues were initially added and assigned according to the WHATIF program web interface [70]. The missing regions were reconstructed and optimized using the template-based loop prediction approach ArchPRED [71]. The side chain rotamers were refined and optimized by the SCWRL4 tool [72]. The protonation states for all the titratable residues of the antibody and RBD proteins were predicted at pH 7.0 using Propka 3.1 software and web server [73,74]. The protein structures were then optimized using atomic-level energy minimization with composite physics and knowledge-based force fields implemented in the 3Drefine method [75,76]. The NAMD 2.13-multicore-CUDA package [77] with CHARMM36 force field [78] was employed to perform 1 µs all-atom MD simulations for the RBD-antibody complexes. The structures of the complexes were prepared in Visual Molecular Dynamics (VMD 1.9.3) [79] with the CHARMM-GUI web server [80,81] using the Solutions Builder tool. Hydrogen atoms were modeled onto the structures prior to solvation with TIP3P water molecules [82] in a periodic box that extended 10 Å beyond any protein atom in the system. To neutralize the biological system before the simulation, Na^+^ and Cl^−^ ions were added in physiological concentrations to achieve charge neutrality, and a salt concentration of 150 mM of NaCl was used to mimic physiological concentration. All Na^+^ and Cl^−^ ions were placed at least 8 Å away from any protein atoms and from each other.

MD simulations are typically performed in an aqueous environment in which the number of ions remains fixed for the duration of the simulation, with a minimally neutralizing ion environment or salt pairs to match the macroscopic salt concentration [83]. All systems were subjected to a minimization protocol consisting of two stages. First, minimization was performed for 100,000 steps of steepest descent with all the hydrogen-containing bonds constrained and the protein atoms fixed. In the second stage, minimization was performed for 50,000 steps using the conjugate gradient approach with all the protein backbone atoms fixed and for an additional 10,000 steps with no fixed atoms. After minimization, the protein systems were equilibrated in steps by gradually increasing the system temperature in steps of 20 K, increasing from 10 K to 310 K, and at each step, a 1 ns equilibration was performed, maintaining a restraint of 10 kcal mol^−1^ Å^−2^ on the protein Cα atoms. After the restraints on the protein atoms were removed, the system was equilibrated for an additional 10 ns. Long-range, non-bonded van der Waals interactions were computed using an atom-based cutoff of 12 Å, with the switching function beginning at 10 Å and reaching zero at 14 Å. The SHAKE method was used to constrain all the bonds associated with hydrogen atoms. The simulations were run using a leap-frog integrator with a 2 fs integration time step. The ShakeH algorithm in NAMD was applied for the water molecule constraints. The long-range electrostatic interactions were calculated using the particle mesh Ewald method [84] with a cut-off of 1.0 nm and a fourth-order (cubic) interpolation. The simulations were performed under an NPT ensemble with a Langevin thermostat and a Nosé–Hoover Langevin piston at 310 K and 1 atm. The damping coefficient (gamma) of the Langevin thermostat was 1/ps. In NAMD, the Nosé–Hoover Langevin piston method is a combination of the Nosé–Hoover constant pressure method [85] and piston fluctuation control implemented using Langevin dynamics [86,87]. An NPT production simulation was run on equilibrated structures for 1 µs, keeping the temperature at 310 K and a constant pressure (1 atm).

### 2.2. Mutational Scanning of the Binding Interfaces for the SARS-CoV-2 S Protein Complexes with Antibodies

To understand the molecular mechanisms underlying the interactions between the SARS-CoV-2 S-RBD and antibodies, we conducted a comprehensive mutational scanning analysis of the binding epitope residues. This approach systematically evaluated the effects of mutations on protein stability and binding free energy, providing insights into the structural and energetic determinants of RBD–antibody interactions. Each binding epitope residue in the RBD-antibody complexes was systematically mutated using all possible amino acid substitutions. The corresponding changes in protein stability and binding free energy were computed using the BeAtMuSiC approach [88,89,90]. This method relies on statistical potential that describe pairwise inter-residue distances, backbone torsion angles, and solvent accessibility. The BeAtMuSiC approach evaluates the impact of mutations on both the strength of interactions at the protein–protein interface and the overall stability of the complex. The binding free energy of a protein–protein complex is expressed as the difference between the folding free energy of the complex and the folding free energies of the individual binding partners:

(1) $$\Delta G_{bind}=G^{com}-G^{A}-G^{B}$$

The change in the binding energy due to a mutation was then calculated as

(2) $$\Delta \Delta G_{bind}=\Delta G_{bind}^{mut}-\Delta G_{bind}^{wt}$$

To ensure robust results, we leveraged rapid calculations based on statistical potentials, computing ensemble-averaged binding free energy changes using equilibrium samples from molecular dynamics (MD) simulation trajectories. The binding free energy changes were averaged over 10,000 equilibrium samples for each system studied. We used 1000 ns of equilibrated trajectory data for each system, with snapshots collected at 100 ps intervals.

### 2.3. Binding Free Energy Computations of the SARS-CoV-2 S Protein Complexes with Antibodies

We employed the Molecular Mechanics/Generalized Born Surface Area (MM/GBSA) method [91,92] for binding free energy calculations of the S-antibody complexes to ensure robust sampling, and equilibrium trajectories were extracted from the production phase of the MD simulations. Specifically, we used 1000 ns of equilibrated trajectory data for each system, with snapshots collected at 100 ps intervals. This approach provided a total of 10,000 frames per system for binding free energy calculations. Additionally, we conducted energy decomposition analysis to evaluate the contribution of each amino acid during the binding of RBD to antibodies [93,94].

The binding free energy for the RBD-antibody complex was obtained using:

(3) $$\Delta G_{\mathrm{bind}}=G_{\mathrm{RBD}-\mathrm{AB}}-G_{\mathrm{RBD}}-G_{\mathrm{AB}}$$

(4) $$\Delta G_{bind,MMGBSA}=\Delta E_{MM}+\Delta G_{sol}-T\Delta S$$

where ΔE_MM_ is the total gas phase energy (sum of ΔEinternal, ΔEelectrostatic, and ΔEvdw), and ΔGsol is the sum of polar (ΔGGB) and non-polar (ΔGSA) contributions to solvation. Here, G_RBD–ANTIBODY_ represents the average over the snapshots of a single trajectory of the complex, and G_RBD_ and G_ANTIBODY_ correspond to the free energy of RBD and the antibody, respectively.

The polar and non-polar contributions to the solvation free energy were calculated using a Generalized Born solvent model and consideration of the solvent accessible surface area [95]. MM-GBSA was employed to predict the binding free energy and decompose the free energy contributions to the binding free energy of a protein–protein complex on a per residue basis. The binding free energy with MM-GBSA was computed by averaging the results of computations over 10,000 samples from the equilibrium ensembles. We used 1000 ns of equilibrated trajectory data for each system, with snapshots collected at 100 ps intervals. This approach provided a total of 10,000 frames per system for binding free energy calculations. The single trajectory protocol uses one trajectory of the RBD-antibody complex, reducing noise by canceling out intermolecular energy contributions. This protocol is suitable when significant structural changes upon binding are not expected. We employed a single-trajectory protocol due to its lower noise and applicability to systems with minimal structural reorganization upon binding. Entropy contributions are typically excluded from calculations because the entropic differences in relative binding affinities are expected to be small for minor mutational changes [96,97]. MM/GBSA calculations were performed using the MMPBSA.py module in AMBER, with the dielectric constant set to 1 for the solute and 80 for the solvent in the AmberTools21 package [98] and gmx_MMPBSA, a new tool to perform end-state free energy calculations from CHARMM and GROMACS trajectories [99].

## 3. Results

### 3.1. Structural Analysis and MD Simulations of the S-RBD Complexes with Antibodies

**Figure 2:**
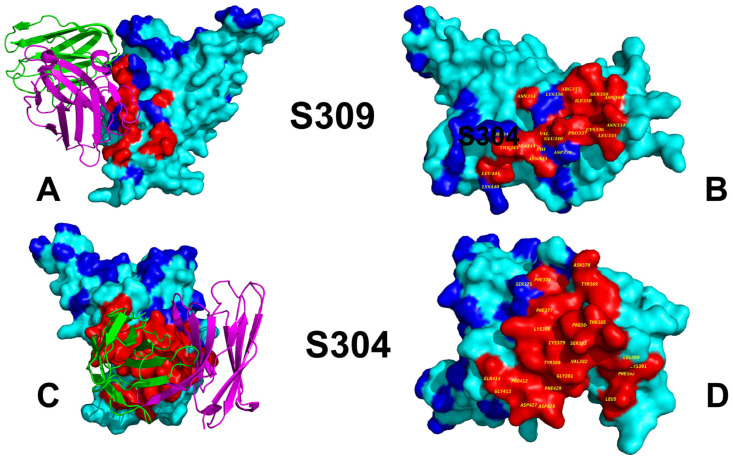
Structural organization of the RBD complexes and binding epitopes of S304 (**A**,**B**) and S304 antibody (**C**,**D**). The B1 RBD complex with S309 (pb id 7YAD) and Wu-WT RBD complex with S304 (pdb id 8WPY) are used for visualization. The S-RBD structure is shown in the cyan surface. The heavy chains of antibodies are in green ribbons and the light chains are in magenta ribbons. (**B**) The RBD surface and binding epitope for S309-RBD complex. RBD is shown in the cyan surface. The binding epitope residues are in the red surface (N334, L335, P337, D339, E340, N343, A344, T345, R346, F347, N354 K356, R357, S359, L441, and K444). (**D**) The RBD surface and binding epitope for S304-RBD complex. RBD is shown in the cyan surface. The binding epitope residues are in the red surface (Y369, N370, F374, F377, K378, C379, Y380, G381, V382, S383, P384, T385, K386, L390, F392, P412, G413, D427, T430, L517). The key sites of Omicron XBB, BA.2.86, and JN.1 lineages are shown in the blue surface (residues 339, 346, 356, 371, 373, 375 376, 403, 405, 408, 417, 440, 444, 445, 446, 450, 452, 455, 456, 460, 475, 477, 478, 481, 484, 486, 493, 498, 501, 505).

All-atom MD simulations were conducted for the S-RBD complexes with a panel of studied antibodies to explore their conformational landscapes and identify specific dynamic signatures induced by antibody binding. The primary objective of this study was to investigate the dynamic and energetic contributions of RBD residues, as these residues play a pivotal role in mediating interactions with both host cell receptors and neutralizing antibodies. The epitope recognized by S309 is located on the outer surface of the RBD, which becomes exposed when the spike protein adopts the “up” conformation required for receptor engagement. This accessibility ensures that S309 can effectively bind to the RBD during viral entry. The S309 epitope involves residues spanning three key regions (Figure 2A,B). By targeting a region near the ACE2-binding site (Figure 2A,B, Supporting Information, Figure S1A), S309 effectively blocks viral entry while maintaining broad-spectrum neutralization activity across multiple variants. In contrast, the S304 antibody recognizes a distinct epitope on the RBD that involves residues from multiple structural elements, including α-helices, β-strands, and flexible loops. This epitope is structurally complex and contributes to the formation of the conserved RBD β-sheet (Figure 2C,D). The S304 epitope comprises residues 369–392, which are part of two α-helices and an intervening β-strand (Figure 2C,D, Supporting Information, Figure S1B). These regions participate in the formation of the structurally conserved RBD β-sheet, underscoring the stability of the interaction. The binding epitope of S304 includes the following key residues, Y369, N370, F374, S375, F377, K378, 379, 380–386, 390, and 392, that form a dense network of interactions, including hydrogen bonds, van der Waals forces, and hydrophobic packing (Supporting Information, Figure S1B).

The involvement of both structured elements (α-helices and β-strands) and flexible loops highlights the dynamic nature of S304 binding. This dual engagement ensures strong and specific interactions, even in the presence of minor mutations in the RBD. While both S309 and S304 are potent neutralizing antibodies, their epitopes differ significantly in terms of location, composition, and functional implications. S309 binds near the ACE2-binding site, allowing it to block receptor engagement through steric hindrance. S304 targets a more peripheral region of the RBD, involving residues that contribute to the structural integrity of the RBD β-sheet. While the S309 epitope involves conserved residues 334-346 and a structural loop (443–450) near the ACE2 interface (Figure 2A,B), the S304 epitope encompasses a broader range of structural elements, including α-helices, β-strands, and flexible loops (Figure 2C,D).

**Figure 3:**
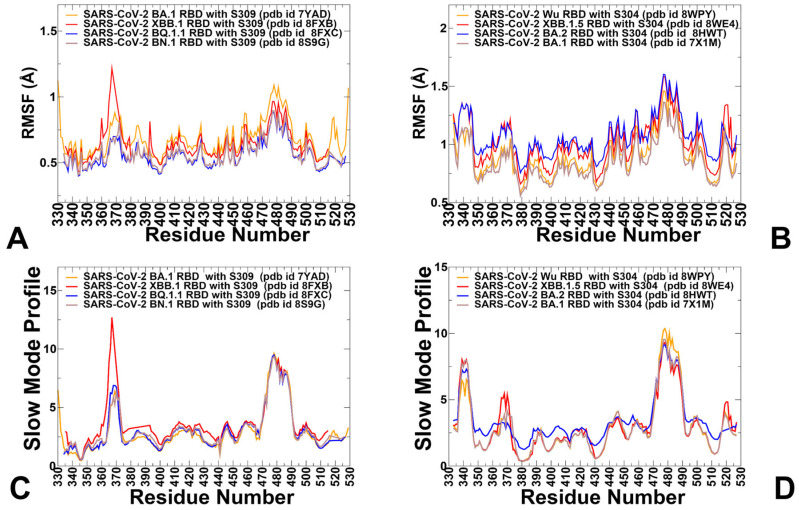
Conformational dynamics profiles obtained from simulations of the RBD-antibody complexes. (**A**) The RMSF profiles for the RBD residues obtained from MD simulations of the S-RBD complexes with S309 using different structures: BA.1 RBD, pdb id 7YAD (in orange lines); XBB.1 RBD, pdb id 8FXB (in red lines); BQ.1.1 RBD, pdb id 8FXC (in blue lines); and BN1RBD, pdb id 8S9G (in light brown lines). (**B**) The RMSF profiles for the RBD residues obtained from MD simulations of the S-RBD complexes with S304 antibody using different structures: Wu-WT, RBD pdb id 8WPY (in orange lines); XBB.1.5 RBD, pdb id 8WE4 (in red lines); BA2 RBD, pdb id 8HWT (in blue lines); and BA.1 RBD, pdb id 7X1M (in light brown lines). (**C**) The slow mode profile averaged over 10 lowest frequency modes obtained from MD simulations of RBD-S309 complexes using different structures: BA.1 RBD, pdb id 7YAD (in orange lines); XBB.1 RBD, pdb id 8FXB (in red lines); BQ.1.1 RBD, pdb id 8FXC (in blue lines); and BN1RBD, pdb id 8S9G (in light brown lines). (**D**) The slow mode profile averaged over the 10 lowest frequency modes obtained from MD simulations of RBD-S304 complexes using different structures: Wu-WT RBD, pdb id 8WPY (in orange lines); XBB.1.5 RBD, pdb id 8WE4 (in red lines); BA2 RBD, pdb id 8HWT (in blue lines); and BA.1 RBD, pdb id 7X1M (in light brown lines).

To characterize the dynamic flexibility of the RBD when bound to different antibody groups, we performed root-mean-square fluctuation (RMSF) analysis on equilibrated MD trajectories (Figure 3). First, we compared the RMSF profiles of the RBD-S309 complexes obtained from MD simulations of four different RBD-S309 structures for the different Omicron variants BA.1, XBB.1, BQ.1.1, and BN.1 (Figure 3A). The RMSF profiles of the RBD-S309 complexes revealed a high degree of similarity across the four Omicron subvariants, reflecting the conserved structural core of the RBD. However, notable differences were observed in specific regions. The central β-sheet and α-helices of the RBD exhibited low RMSF values, indicating minimal flexibility. These regions are critical for maintaining the overall structural integrity of the RBD. Two key regions displayed higher flexibility. Residues 355–375 showed moderate fluctuations, likely due to their proximity to the S309 epitope and involvement in mediating interactions with the antibody. In the RMSF profiles, the 470–490 loop consistently showed elevated flexibility across all four variants, although subtle differences were observed. The XBB.1 and BQ.1.1 variants displayed higher flexibility, suggesting that mutations in these subvariants may enhance the loop’s adaptability to accommodate antibody binding.

The S304 antibody interacts with specific regions of the RBD, including residues 369–392 (two α-helices and an intervening β-strand), residues 515–517 (part of the structurally conserved RBD β-sheet), and loop residues 411–414 and 427–430. The RMSF values for residues 369–392 are relatively low, indicating minimal flexibility (Figure 3B). These residues form the core of the epitope and play a pivotal role in stabilizing the interaction between S304 and the RBD. Residues Y369, N370, F374, S375, F377, K378, 379, 380–386, 390, and 392 form a dense network of interactions, including hydrogen bonds, van der Waals forces, and hydrophobic packing. These interactions further reduce flexibility and enhance binding affinity. The RMSF values for loop residues 411–414 and 427–430 are higher compared to the structured core, indicating significant flexibility. This flexibility allows these loops to adjust their conformation to optimize interactions with the antibody. The loops play a crucial role in mediating antibody recognition by forming hydrogen bonds and van der Waals contacts with S304. Their adaptability ensures that the interaction remains stable even in the presence of minor mutational changes. S309 binding stabilizes the core of the RBD, locking it into a rigid conformation that is less conducive to large-scale motions. This stabilization indirectly restricts the flexibility of the 470–490 loop. S304 engages a broader and more diverse set of residues, requiring the RBD to adopt a more dynamic conformation. This leads to increased flexibility in the 470–490 loop to accommodate the antibody’s binding requirements. The increased flexibility of the 470–490 loop in the S304-bound state highlights its role in adaptive binding (Figure 3A,B). This adaptability ensures that S304 can effectively neutralize diverse variants despite antigenic drift. Conversely, the reduced flexibility of the loop in the S309-bound state reflects the antibody’s ability to stabilize the RBD in a conformation that sterically hinders ACE2 binding, thereby preventing viral entry.

We also characterized essential motions and determined the hinge regions in the RBD-S309 and RBD-S304 complexes (Figure 3C,D) using the PCA of trajectories in the CARMA2.3 package [100]. The local minima along these profiles are typically aligned with the immobilized global motions hinge centers, while the maxima correspond to the moving regions undergoing concerted movements leading to global changes in the structure. The low-frequency soft modes are characterized by their cooperativity, and there is a strong relationship between conformational changes and the “soft” modes of motions intrinsically accessible to protein structures [101,102]. The slow mode profile computed by averaging essential motions over the ten lowest modes revealed key hinge positions corresponding to T346, F375, L441, and D467 residues (Figure 3C). Group E antibodies are particularly sensitive to mutations at residues G339, T345, and R346, which are located near the N-terminal region of the RBD. These residues play a critical role in stabilizing the RBD structure and facilitating interactions with certain antibodies. L441 is central to the interaction, forming hydrogen bonds and van der Waals contacts with S309. The regions that undergo large movements in slow modes are regions 362–375 and 470–490 (Figure 3C). The stabilization of the RBD core and reduction in flexibility highlight the robust nature of S309 binding and its effectiveness in neutralizing the virus. In contrast to the RBD-S309 complex, the slow mode profile of the RBD-S304 complex features a more dynamic behavior, characterized by hinge regions that are aligned with positions 374–385 and 429–436, indicating a broader distribution of pivot points compared to the RBD-S309 complex (Figure 3D). Several regions exhibit enhanced mobility in the presence of S304, including residues 360–380, 430–450, and 468–494. The RBD-S304 complex exhibits more fluctuating behavior in global modes, as evidenced by a more distributed pattern of motion across multiple regions (Figure 3D). Enhanced flexibility is observed in regions such as 360–380 and 430–450, indicating that these segments undergo coordinated structural adjustments during S304 binding.

**Figure 4:**
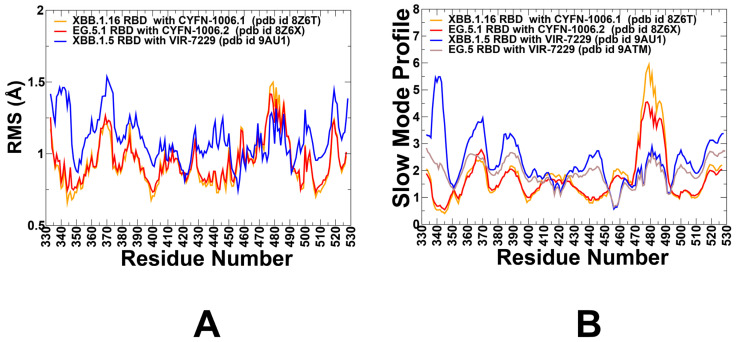
Conformational dynamics profiles obtained from simulations of the RBD-antibody complexes. (**A**) The RMSF profiles for the RBD residues obtained from MD simulations of the S-RBD complexes with CYFN-1006.1 (XBB.1.16 RBD, pdb id 8Z6T, in orange lines), CYFN-1006.2 (EG.5.1 RBD, pdb id 8Z6X, in red lines), and VIR-7229 (XBB.1.5 RBD, pdb 9AU1, in blue lines). (**B**) The slow mode profiles for the RBD residues obtained by averaging over the 10 lowest frequency modes from MD simulations of the S-RBD complexes with CYFN-1006.1 (XBB.1.16 RBD, pdb id 8Z6T, in orange lines), CYFN-1006.2 (EG.5.1 RBD, pdb id 8Z6X, in red lines), VIR-7229 (XBB.1.5 RBD, pdb 9AU1, in blue lines), and VIR-7229 (EG.5 RBD, pdb id 9ATM, in brown lines).

CYFN-1006 binds to a distinct and structurally conserved epitope on the RBD encompassing residues 339–348, 354–356, 399, 440–446, 450, and 499–500. CYFN-1006.1 engages a large and more distributed interface involving 17 RBD residues spanning multiple structural elements, including T345, N343, A344, K440, T346, P445, and L441 (Supporting Information, Figure S1C). Key conserved residues—N343, A344, and T345—form hydrogen bonds that anchor the interaction, contributing significantly to binding stability (Supporting Information, Figure S1C). Additional hydrophobic contacts involving K440, L441, and P445 further stabilize the binding interface [41]. These interactions collectively support the broad neutralization profile of CYFN-1006 across multiple Omicron variants. The RMSF profiles for the RBD in complex with CYFN-1006 revealed that residues 339–348 form part of the conserved core epitope and exhibit low RMSF values, indicating minimal flexibility. Residues 354–356 show moderate flexibility, likely due to their proximity to the antibody’s complementarity-determining regions (CDRs) and involvement in mediating interactions with CYFN-1006. Residues 440–446 exhibit low RMSF values, reflecting the effect of binding. The flexible loop region 470-490 continues to be the most dynamic region in the complex (Figure 4A). VIR-7229 targets a structurally complex epitope on the RBD that includes residues 403–417, 453–460, and 473–477, among others [42]. These regions contribute to both hydrophobic core formation and shape complementarity at the binding interface. Notably, 13 of the 25 epitope residues overlap with the ACE2-binding interface, highlighting the antibody’s ability to block receptor engagement. The involvement of this epitope is reflected in the RMSF analysis (Figure 4A), which shows moderate flexibility for residues 403–417, consistent with their engagement in VIR-7229 binding. The region spanning 425–460 exhibits elevated fluctuations, indicating dynamic behavior that may facilitate dual interactions with both antibodies and ACE2. Similarly, loop residues 473–477 display moderately increased mobility, aligning with structural studies that report conformational rearrangements upon VIR-7229 binding [42]. The analysis of slow mode profiles revealed the unique feature of functional motions for the VIR-7229 complex (Figure 4B). In this case, the moving regions correspond to residues 355–375, while the rest of the RBD residues experience only small movements, including residues 470–490. A unique feature of VIR-7229 binding is its reliance on backbone-mediated interactions. This characteristic is reflected in the slow mode profiles of the VIR-7229 complex with RBD, as hydrogen bonds formed between VIR-7229 and the RBD involve backbone atoms of residues such as N417, L455, R457, and K458. These positions and regions 417–422 and 455–460 become associated with the broad hinge points anchoring the RBD in the complex with VIR-7229 (Figure 4B).

The slow mode analysis revealed that VIR-7229 immobilizes key regions of the RBD, including residues 455–460, locking F456 or L456 in the optimal binding position and preserving the character of functional motions. These hinge points anchor the RBD and stabilize the interaction. Despite the F456L mutation in EG.5, the slow mode profile of the RBD-VIR-7229 complex remains largely unchanged. We also observed that RBD regions 350–370 and 38–395 may undergo appreciable displacements in global motions, indicating the ability of dynamic adjustments of the RBD to bind VIR-7229 (Figure 4B). The region 455–460 continues to exhibit reduced flexibility, indicating that the antibody’s binding mode accommodates both F456 and L456 without compromising stability or altering the location of the hinge points (Figure 4B). Structural studies showed that VIR-7229 makes persistent contacts with both F456 (XBB.1.5) and L456 (EG.5) [42] and therefore can be effective against Omicron variants featuring mutations in these positions. The ability of VIR-7229 to tolerate both F456 (found in XBB.1.5) and L456 (found in EG.5) can be attributed to its unique binding mechanism, which relies on backbone-mediated interactions. These findings highlight the adaptability of VIR-7229 to mutations of convergent evolution hotspots while also underscoring the nuanced impact of such mutations on RBD dynamics.

### 3.2. Mutational Profiling of Protein Binding Interfaces

**Figure 5:**
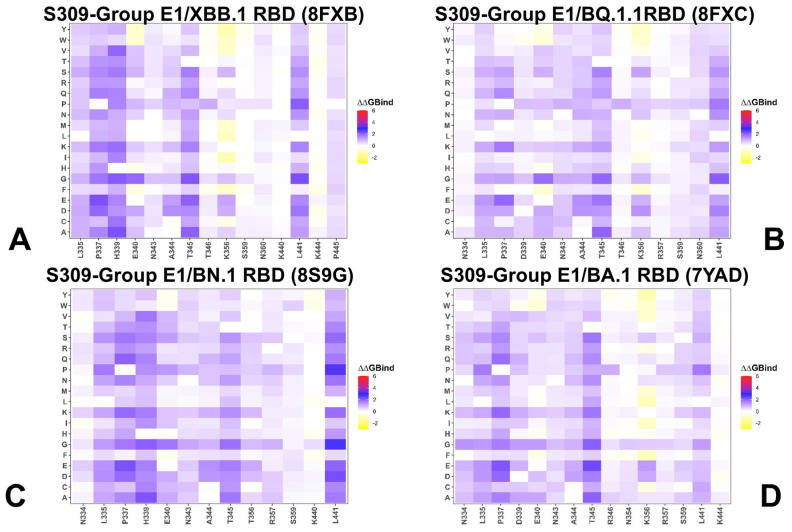
The ensemble-based mutational scanning of binding for the SARS-CoV-2 S-RBD complexes with S309 antibody. The mutational scanning heatmaps for the binding epitope residues in the S-RBD complexes with XBB.1 RBD, pdb I 8FXB (**A**); BQ.1.1 RBD, pdb id 8FXC (**B**); BN.1 RBD, pdb id 89GG (**C**); and BA.1 RBD, pdb id 7YAD (**D**). The binding energy hotspots correspond to residues with high mutational sensitivity. The heatmaps show the computed binding free energy changes for 20 single mutations on the sites of variants. The squares on the heatmap are colored using a three-colored blue-white-yellow scale, with yellow indicating the largest unfavorable effect on stability. The standard errors of the mean for binding free energy changes were based on 500 samples from the MD trajectory within 0.08–0.15 kcal/mol.

Using the conformational ensembles of the RBD complexes, we embarked on structure-based mutational analysis of the S protein binding with a panel of antibodies under investigation in this study. To provide a systematic comparison, we constructed mutational heatmaps for the RBD interface residues of the S complexes with S309, S304, CYFN1006-1, CYFN1006-2, and VIR-7229 antibodies. The mutational scanning of the RBD residues in complexes with the S309 antibody provides critical insights into the structural and functional determinants of their interaction. Mutational scanning identified the following residues as critical for binding the S309 antibody: N334, L335, P337, D339, T345, R346, and L441 (Figure 5, Supporting Information, Datasets S1–S4). N334 is part of the helical region (residues 337–344) that interacts with the complementarity-determining region (CDR) loops of S309. Specifically, N334 forms hydrogen bonds with the S309 paratope, stabilizing the interaction. Substitutions at N334 could disrupt these hydrogen bonds, reducing the binding affinity of S309. This makes N334 a critical residue for maintaining high-affinity interaction. L335 contributes to hydrophobic interactions with the S309 antibody. Hydrophobic interactions are essential for burying the surface area at the antibody–RBD interface, ensuring tight binding. Mutations at L335 could introduce polar or charged residues, disrupting the hydrophobic core and destabilizing the interaction. P337 is located within the helical region and plays a structural role in maintaining the conformation of the epitope. Proline residues often induce kinks or turns in secondary structures, which can influence the accessibility of the epitope. Substitutions at P337 could alter the local conformation of the helix, potentially affecting the orientation of nearby residues and impairing S309 binding.

Our results are consistent with the experiments that showed S309 binding is largely maintained against Omicron variants except the BA.2.75.2, CA.3, CH.1.1, BA.2.86, and JN.1 variants. CH.1.1 acquired the R346T and F486S mutations present in BA.2.75.2 as well as harbored the additional K444T and L452R. The CA.3.1 variant acquired R346T, F486S, K444M, and L452R mutations [103]. This study showed that R346T could confer strong neutralization resistance to S309-like antibodies in CH.1.1 and CA.3.1 [103]. A series of SARS-CoV-2 variants with mutations at the L455, F456, and R346 positions include the “SLip” variant, which carries L455S along with an additional F456L mutation. More recently, the “FLiRT” variant has appeared, featuring an additional R346T mutation on the SLip backbone. Studies have shown that the SLip and FLiRT subvariants of JN.1 exhibited a complete escape of neutralization by S309 [104]. Residues D339 and R346 lie within the epitope region of S309. Mutations at these positions are present in lineages FLiRT and KP.2, which could enhance viral evasion from antibody neutralization. Several studies showed S309 maintained efficacy against Omicron variants, including BA.2.87.1, with the exception of CH.1.1, CA.3.1, BA.2.75.2, and BA.2.86 [103,104,105]. The BA.2.87.1 variant features N417T, K444N, V445G, L452M, K478T, N481K, and R493Q [105] and can be effectively neutralized by S309. Across all four S309-RBD complexes, the T345, R346, and L441 positions correspond to mutational hotspots and variations in these sites; particularly, the R346T convergent mutation can render resistance to S309 binding (Figure 5, Supporting Information, Datasets S1–S4). Mutational scanning also revealed the P337 position as an important hotspot for S309 binding (Figure 5, Supporting Information, Datasets S1–S4). This is consistent with reported data that any substitution of the helix breaker P337 resulted in a complete S309 escape [106].

**Figure 6:**
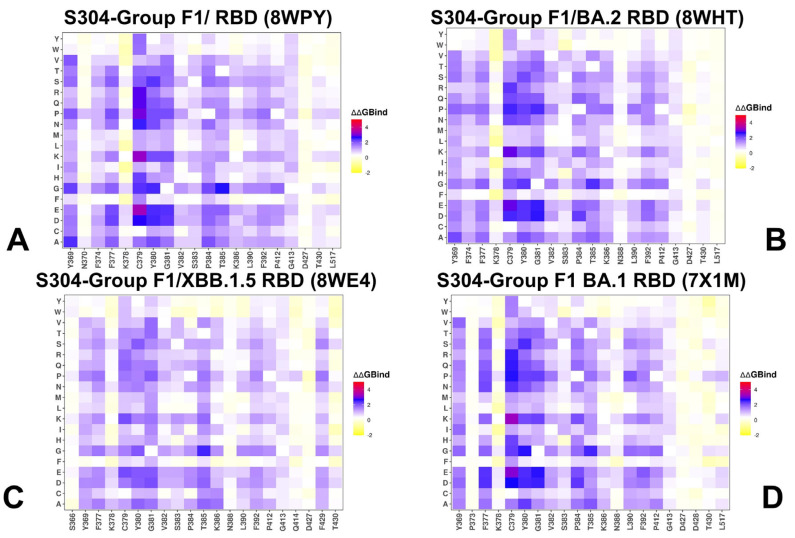
The ensemble-based mutational scanning of binding for the SARS-CoV-2 S-RBD complexes with S304 antibody. The mutational scanning heatmaps for the binding epitope residues in the S304 complexes with Wu-WT RBD, pdb id 8WPY (**A**); BA.2 RBD, pdb id 8HWT (**B**); XBB.1.5 RBD, pdb id 8WE4 (**C**); and BA.1 RBD, pdb id 7X1M (**D**). The binding energy hotspots correspond to residues with high mutational sensitivity. The heatmaps show the computed binding free energy changes for 20 single mutations on the sites of variants. The squares on the heatmap are colored using a three-colored blue-white-yellow scale, with yellow indicating the largest unfavorable effect on stability. The standard errors of the mean for binding free energy changes were based on 500 selected samples from the MD trajectory within 0.12 kcal/mol.

Mutational scanning of the RBD in complexes with the S304 antibody across four different structures revealed consistent hotspots that play critical roles in stabilizing the interaction. These hotspots include residues Y369, F374, F377, C379, Y380, G381, V382, S383, P384, T385, F390, and L392. Among these, S383, P384, and T385 emerged as the most significant energy hotspots (Figure 6, Supporting Information, Datasets S5–S8). Mutational scanning identifies T385 as the most critical residue for binding of S304, showing high sensitivity to substitutions that significantly reduce binding affinity [27,39,40]. This makes T385 a key mutational hotspot and a likely target for immune evasion. Other residues such as S383 and P384 also contribute significantly to the binding interface, with mutational profiles indicating their importance in maintaining epitope integrity. Additionally, Y380 shows sensitivity to mutation, and Y380Q may interfere with the binding of neutralizing antibody S304 [107]. K386 plays a crucial role in electrostatic stabilization, working synergistically with T385 to enhance binding affinity. The deleterious effects of mutations K386D/R indicate that disruptions to this electrostatic network can significantly impair S304 binding [27,39,40]. These findings from mutational heatmaps are consistent with DMS data highlighting the vulnerability of this epitope to amino acid substitutions and providing insights into mechanisms by which the virus may evolve to escape recognition by S304.

**Figure 7:**
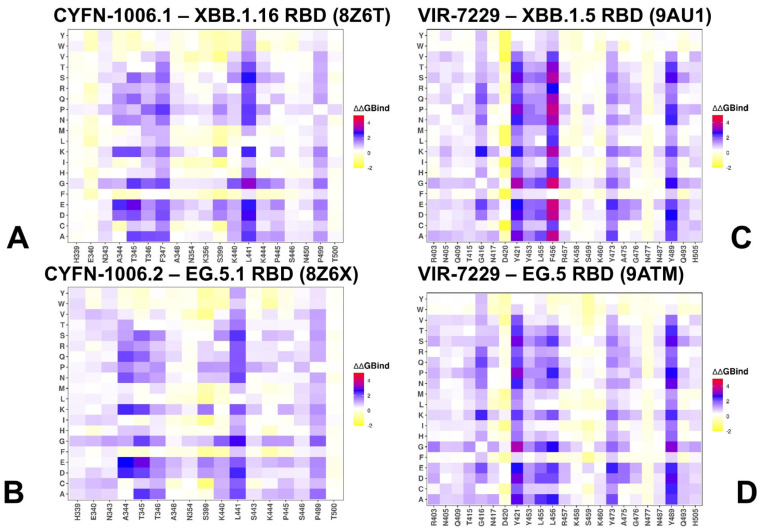
The ensemble-based mutational scanning of binding for the SARS-CoV-2 S-RBD complexes with CYFN-1006 (**A**,**B**) and VIR-7229 (**C**,**D**). The mutational scanning heatmaps for the binding epitope residues in the CYFN-1006.1 complex with XBB.1.16 RBD, pdb id 8Z6T (**A**) and CYFN-1006.2 complex with EG.5.1 RBD, pdb id 8Z6X (**B**). The heatmaps for VIR-7229 complexes with XBB.1.5 RBD, pdb id 9AU1 (**C**) and EG.5 RBD, pdb id 9ATM (**D**). The binding energy hotspots correspond to residues with high mutational sensitivity. The heatmaps show the computed binding free energy changes for 20 single mutations on the sites of variants. The squares on the heatmap are colored using a three-colored blue-white-yellow scale, with yellow indicating the largest unfavorable effect on stability. The standard errors of the mean for binding free energy changes were based on 500 selected samples from the MD trajectory within 0.21 kcal/mol.

Mutational scanning of the RBD residues in complexes with CYFN-1006.1 and CYFN-1006.2 antibodies (Figure 7A,B, Supporting Information, Datasets S9 and S10) consistently unveiled key energetic hotspots at positions A344, T345, T346, F347, L441, and P499. Importantly, there are no notable Omicron variants that carry mutations in positions A344, T345, F347, L441, and P499. However, a newly discovered point R346X, primarily R346T, is connected with a surge in SARS-CoV-2 infections, as the R346T mutation is predominantly expressed in many Omicron subvariants. Generally, the three notable amino acid substitutions at the R346 position of the spike RBD are R346K, R346T, and R346I. Most of the offspring descended from the Omicron sublineages, including BJ.1, BR.3, and BA.2.75.5, show a strong predominance of R346T [108]. R346K is in B1.62.1 and BA.1.1 [109]. Notably, we used an ensemble from simulations of XBB.1.16 and EG.5.1 RBD in complexes with antibodies CYFN1006-1/2, in which the positions T345 and T346 are present. It appears that mutations in the T346 position can significantly moderately reduce binding, and R346T is present in these variants as well as in KP.2 but not in JN.1 and KP.3 (Figure 7A,B, Supporting Information, Datasets S9 and S10). CYFN1006-1 was tested against B.1.1.7, B.1.351, P.1, B.1.617.2, BA.1, and Variants of Interest (VOIs) (B.1.525, B.1.621, C.37) as well as various XBB subvariants, along with recently identified and currently circulating variants such as JN.1, KP.2, KP.3, KP.3.1.1, and XEC [41].

Of particular interest are mutational heatmaps of VIR-7229 binding to XBB.1.5 RBD (Figure 7C, Supporting Information, Dataset S11) and EG.5 RBD (Figure 7D, Supporting Information, Dataset S12). These maps are highly similar and point to mutational hotspots at positions G416, Y421, Y453, L455, F456/L456, Y473, and Y489. Hence, despite a large binding interface, a hydrophobic group of highly conserved central residues is associated with VIR-7229 binding. With the notable exception of the L455 and F456 positions, other residues have a high mutational barrier and are typically not observed in Omicron variants. KP.2 carries mutations R346T and F456L, and KP.3 features mutations R346T, L455S, F456L, and Q493E. KP.3 has emerged as the most immune-evasive and fastest-growing JN.1 sublineage, largely due to the F456L mutation, which plays a critical role in antibody escape [63]. Mutational heatmaps showed that F456 can be more favorable for binding, while L456 mutations are somewhat better tolerated but still highlight the binding hotspot for both F456 and L456 (Figure 7C,D, Supporting Information, Datasets S11 and S12).

Our results are in line with the DMS experiments that exhaustively mapped the escape profile of the Wuhan-Hu-1, BA.2, BQ.1.1, XBB.1.5, EG.5, and BA.2.86 yeast-displayed RBDs. VIR-7229 featured a remarkably narrow escape profile in all backgrounds, with only some mutations at F456 causing escape [42]. A direct comparison with mutational escape in the XBB.1.5 background featured in the structure of VIR-7229 (Figure 7C, Supporting Information, Dataset S11), and the mutations causing significant escape are F486K/E/P [42]. Our results showed that the largest destabilization free energies were for mutations of F456A (ΔΔG = 3.75 kcal/mol), F456D (ΔΔG = 3.74 kcal/mol), F456K (ΔΔG = 3.49 kcal/mol), F456E (ΔΔG = 3.94 kcal/mol), and F486P (ΔΔG = 3.82 kcal/mol) (Figure 7C, Supporting Information, Dataset S11). More tolerant changes are evident for VIR-7229 binding with EG.5 RBD, which featured F456L modification (Figure 7D, Supporting Information, Dataset S12). Interestingly, mutations L456P and L456S emerged as highly destabilizing, which is consistent with the experiments. To conclude, mutational scanning identified specific residues that are crucial for antibody binding, particularly showing that mutations R346T and F456L are associated with reduced antibody efficacy across multiple variants. These mutations highlight the evolutionary pressure on the virus to evade immune responses.

### 3.3. MM-GBSA Analysis of the Binding Affinities

**Figure 8:**
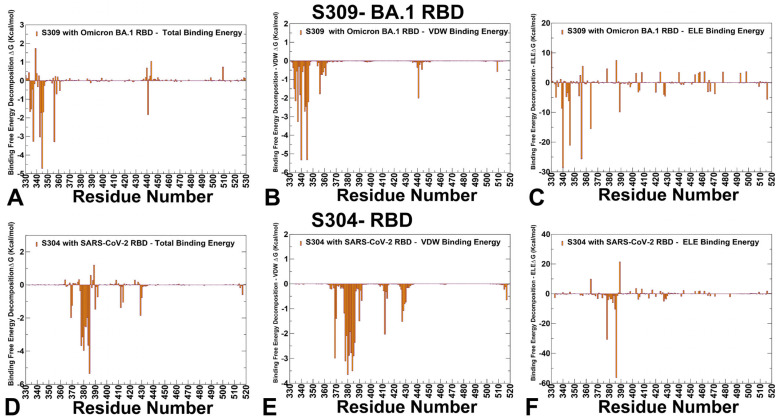
The residue-based decomposition of the binding MM-GBSA energies for S309 and S304 binding. The residue-based decomposition for S309-RBD binding shows the total binding energy (**A**), van der Walls contribution to the total MM-GBSA binding energy (**B**), and the electrostatic contribution to the total binding free energy (**C**). MM-GBSA contributions are evaluated using 1000 samples from the equilibrium MD simulations of S309 complex with BA.1 RBD complex, pdb id 7YAD. The residue-based decomposition for S304-RBD binding shows the total binding energy (**D**), van der Walls contribution to the total MM-GBSA binding energy (**E**), and the electrostatic contribution to the total binding free energy (**F**). MM-GBSA contributions are evaluated using 1000 samples from the equilibrium MD simulations of S304 complex with Wu-WT RBD, pdb id 8WPY.

Using the conformational equilibrium ensembles to obtain MD simulations, we computed the binding free energies for the RBD-antibody complexes using the MM-GBSA method [105,106,107,108]. We examine whether the binding affinities and contributions of the major binding hotspots are largely determined by the van der Waals or electrostatic interactions and whether positions of immune escape can be associated with the binding hotspots where different energetic contributions can act synergistically, leading to significant loss of binding upon mutations. MM-GBSA analysis of S309 antibody complexes and residue decomposition of the total energy (Supporting Information, Tables S2–S5) revealed strong and consistent binding hotspots for the T345(ΔG = −4.72 kcal/mol), K356 (ΔG = −3.29 kcal/mol), L441 (ΔG = −2.83 kcal/mol), N343 (ΔG = −3.03 kcal/mol), A344 (ΔG = −1.72 kcal/mol), and R346 positions (ΔG = −1.69 kcal/mol) (Figure 8A, Supporting Information, Tables S2–S5). This is consistent with mutational scanning computations that identified these residues as critical for binding the S309 antibody. The MM-GBSA residue decomposition analysis further underscores the critical role of L441 and K444 in stabilizing the S309-RBD complex. The energy decomposition showed that the strongest van der Waals interactions are provided by T345, P337, A344, L335, and L441 RBD residues (Figure 8B, Supporting Information, Tables S2–S5). T345 and P337 are key hotspots that are driven by favorable hydrophobic contacts. The largest electrostatic interactions are formed by residues E340, K356, R346, D389, and K444 (Figure 8C, Supporting Information, Tables S2–S5). In particular, K444 contributes primarily through electrostatic stabilization, contributing favorably to the overall electrostatic component of the binding free energy (Figure 8C). L441 and K444 are embedded within the conserved epitope targeted by S309 and are consistently identified as energetic hotspots across multiple Omicron variant complexes. The results indicate that the emergence of the T345, K356, and R346 positions as key hotspots may arise from synergistic favorable van der Waals and electrostatic interactions, whereas the P337 and L441 positions are largely involved in packing interactions. These findings can explain why R346S in combination with P337L enhanced resistance to S309 [34]. R346K in VOC Omicron BA.1.1 reduced sensitivity to S309 [35]. The experiments showed that neutralization activity against BQ, CH, and XBB showed reduced S309 binding and complete loss of binding for the JN.1, KP.2, KP.3, and XEC variants, all of which share the K356T mutation [103,104,105].

The binding free energy analysis of the S304 binding revealed S383, T385, Y380, K378, P384, C379, and V382 as most significant energy hotspots, with T385 (ΔG = −5.36 kcal/mol) emerging as a dominant energetic center (Figure 8D, Supporting Information, Table S6). While van der Waals interactions are strongest for Y380, P384, Y369, and T385, the electrostatic favors K386, K378, and also T385 (Figure 8E, Supporting Information, Table S6). Strikingly, the experimental data on escape hotspots singled out the S383, T385, and K386 sites [27,39,40]. Interestingly, both van der Waals and electrostatic interactions work synergistically to yield favorable binding for T385 and K386 (Figure 8F, Supporting Information, Table S6). These findings are also consistent with mutational scanning computations yielding correct immune hotspots and reproducing DMS data [27]. S383, P384, and T385 emerged as the most significant energy hotspots based on escape maps and binding free energy analysis. T385 consistently emerged as the dominant energetic center, with a binding free energy contribution of ΔG = −5.36 kcal/mol. It is stabilized by both strong van der Waals interactions and electrostatic forces, making it indispensable for S304 binding. S383 and P384 contribute significantly to the stability of the complex through van der Waals interactions, with P384 playing a structural role due to its rigidity, likely influencing the local conformation of the epitope. Hence, binding of S309 is predominantly driven by a combination of hydrophobic packing and electrostatic interactions. Key residues like T345, P337, L441, and R346 play dual roles in stabilizing the complex through van der Waals and electrostatic forces. The synergy between these interactions explains why mutations at R346 (e.g., R346T/K/S) confer resistance to S309. S304 binding is more localized around specific residues, with T385 emerging as the dominant energetic hotspot. Both van der Waals, and electrostatic interactions contribute synergistically to stabilizing the complex. Mutations at T385 and K386 are highly deleterious, indicating their critical roles in maintaining binding affinity.

**Figure 9:**
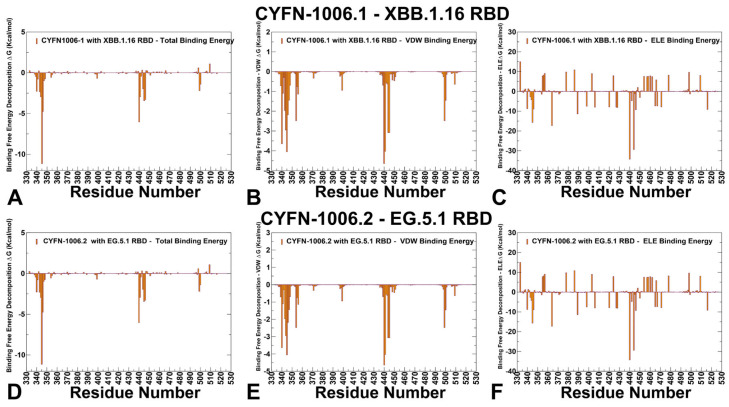
The residue-based decomposition of the binding MM-GBSA energies for CYFN-1006 binding. The residue-based decomposition for CYFN-1006.1 -XBB.1.16 RBD binding shows the total binding energy (**A**), van der Walls contribution to the total MM-GBSA binding energy (**B**), and the electrostatic contribution to the total binding free energy (**C**). MM-GBSA contributions are evaluated using 1000 samples from the equilibrium MD simulations of CYFN-1006.1 complex with XBB.1.16 RBD complex, pdb id 8Z6T. The residue-based decomposition for CYFN-1006.2 -EG.5.1 RBD binding shows the total binding energy (**D**), van der Walls contribution to the total MM-GBSA binding energy (**E**), and the electrostatic contribution to the total binding free energy (**F**). MM-GBSA contributions are evaluated using 1000 samples from the equilibrium MD simulations of C YFN-1006.2 complex with EG.5.1 RBD, pdb id 8Z6X.

CYFN1006-1 largely overlaps with S309 situated on the outer surface of RBD near the RBM region with the epitopes comprising N343, T345, R346, and K356 in the S309 epitope, forming numerous hydrogen bonds and displaying high conservation. The binding affinity of CYFN-1006.1 and CYFN-1006.2 antibodies is driven primarily by N343, A344, T345, K440, T346, P445, S446, and L441 (Figure 9, Supporting Information, Tables S7 and S8). The van der Waals interactions are most favorable for K440, T345, and L441, while the electrostatic favors K440, K444, D364, T365, and D389 (Figure 9B,E, Supporting Information, Tables S7 and S8). Importantly, the K356 position does not provide significant contribution to binding of these antibodies, which may provide enhanced neutralization potency against the JN.1, KP.2, and KP.3 variants sharing the K356T mutation. N343, A344, and T345 play an important role in stabilizing CYFN1006-1 binding to RBD and are highly conserved in various mutant strains. K440, L441, P445, and P499 of RBD are embedded in the hydrophobic patch formed by CDRH1 (Y36, Y37, W38) and CDRH3 (W112) to further stabilize the CYFN1006-1 binding to RBD.

**Figure 10:**
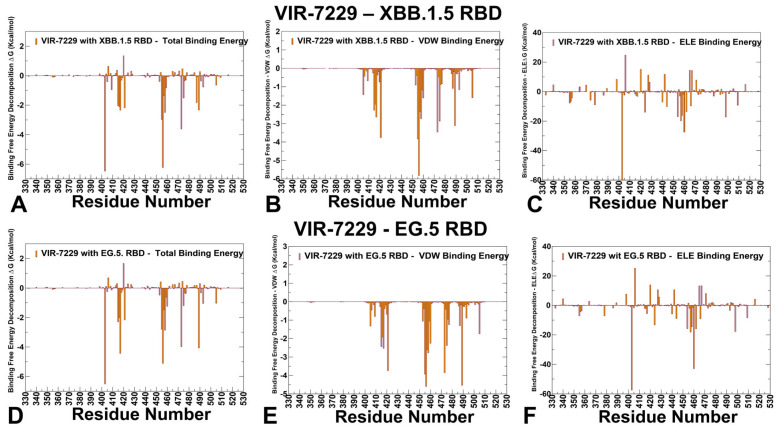
The residue-based decomposition of the binding MM-GBSA energies for VIR-7229 binding. The residue-based decomposition for VIR-7229 -XBB.1.5 RBD binding shows the total binding energy (**A**), van der Walls contribution to the total MM-GBSA binding energy (**B**), and the electrostatic contribution to the total binding free energy (**C**). MM-GBSA contributions are evaluated using 1000 samples from the equilibrium MD simulations of VIR-7229complex with XBB.1.5 RBD complex, pdb id 9AU1. The residue-based decomposition for VIR-7229—EG.5 RBD binding shows the total binding energy (**D**), van der Walls contribution to the total MM-GBSA binding energy (**E**), and the electrostatic contribution to the total binding free energy (**F**). MM-GBSA contributions are evaluated using 1000 samples from the equilibrium MD simulations of VIR-7229 complex with EG.5 RBD, pdb id 9ATM.

A direct comparison with mutational escape in the XBB.1.5 background featured in the structure of VIR-7229; the mutations causing significant escape are F486K/E/P [42]. This may explain why VIR-7229 interactions can tolerate epitope variability, exhibiting a high barrier for the emergence of resistance, partly attributed to its high binding affinity [43]. In particular, VIR-7229 showed neutralization efficacy against KP.2 that carries the mutations R346T and F456L and KP.3 that features the mutations R346T, L455S, F456L, and Q493E. Although R403 and F456L mutations are present in KP.2 and KP.3 variants and arguably can reduce the neutralization potency of VIR-7229, their effect is relatively forgiving. Out of the 11 mutated residues in BA.2.86 RBD relative to XBB.1.5, only R403K is found in the VIR-7229 epitope. Our results suggest that R403K preserves electrostatic interactions with the VIR-7229 light-chain N33 and D52 side chains (Figure 10C,F, Supporting Information, Tables S9 and S10). The JN.1 variant harbors the immune-evasive L455S mutation and is compatible with the VIR-7229 paratope interface due to the small size of the introduced serine side chain. Consistent with these results, mutational scanning of the RBD residues in complexes with CYFN-1006.1 and CYFN-1006.2 antibodies showed key energetic hotspots at positions A344, T345, T346, F347, L441, and P499. Similarly to S309, these antibodies target T345 and T346 sites as dominant hotspots. Even though the binding epitope of CYFN-1006-1 includes 17 RBD residues, the conserved residue T345 along with K440 and T346 anchor the binding affinity of these potent neutralizing antibodies. However, according to the experiments, neutralization activity against BQ, CH, XBB, JN.1, KP.2, KP.3, and XEC variants showed reduced binding or complete loss of binding for S309, while CYFN1006-1 demonstrated consistent neutralization of all tested SARS-CoV-2 variants with unaffected potency. The binding affinity of VIR-7229 to XBB.1.5 RBD showed hotspots in positions R403 (ΔG = −6.47 kcal/mol), F456 (ΔG = −6.23 kcal/mol), Y473 (ΔG = −3.61 kcal/mol), and L455 (ΔG = −2.97 kcal/mol) (Figure 10A, Supporting Information, Table S9). The most favorable van der Waals contacts are made by the F456 (ΔG = −5.82 kcal/mol), L455, Y421, Y473, and Y489 residues, while the electrostatic is most favorable for R403, K460, R457, and R498 (Figure 10B, Supporting Information, Table S9). Interestingly, VIR-7229 binding to EG.5 variant with F456L mutation preserves the ranking of major hotspots R403, L456, 417, Y489, Y473, K458, and L455 (Figure 10D, Supporting Information, Table S10), as L456 makes favorable contacts, allowing for optimal complementarity with L456 (ΔG = −5.11 kcal/mol). The van der Waals interactions for L456 (ΔG = −4.6 kcal/mol) are only moderately weaker than for F456, but the overall binding contribution of mutated F456L continues to be highly favorable (Figure 10E, Supporting Information, Table S10).

A direct comparison with mutational escape in the XBB.1.5 background featured in the structure of VIR-7229; the mutations causing significant escape are F486K/E/P [42]. This may explain why VIR-7229 interactions can tolerate epitope variability, exhibiting a high barrier for the emergence of resistance, partly attributed to its high binding affinity [43]. In particular, VIR-7229 showed neutralization efficacy against KP.2, which carries the mutations R346T and F456L, and KP.3, which features the mutations R346T, L455S, F456L, and Q493E. Although the R403 and F456L mutations are present in KP.2 and KP.3 variants and arguably can reduce the neutralization potency of VIR-7229, their effect is relatively forgiving. Our results suggest that R403K preserves electrostatic interactions with the VIR-7229 light-chain N33 and D52 side chains (Figure 10C,F, Supporting Information, Tables S9 and S10).

In summary, the binding affinity of CYFN1006 is anchored by highly conserved residues such as T345, K440, and T346, which are critical for stabilizing the interaction through both van der Waals and electrostatic forces. Notably, K356 does not significantly contribute to binding, which may explain CYFN1006’s enhanced neutralization potency against variants carrying the K356T mutation. VIR-7229 targets a distinct set of residues, with R403, F456, Y473, and L455 emerging as dominant energetic hotspots. The antibody relies on a combination of hydrophobic and electrostatic interactions, with F456 playing a central role in hydrophobic packing and R403 contributing strongly to electrostatic stabilization. While residues F456 and L455 are critical for binding, VIR-7229’s adaptability to mutations at these positions suggests a broader tolerance for evolutionary changes.

## 4. Discussion

The results presented in this study provide a comprehensive understanding of the molecular mechanisms underlying the interactions between SARS-CoV-2 S-RBD and neutralizing antibodies, including S309, S304, CYFN1006, and VIR-7229. These findings reveal key insights into the structural, energetic, and mutational determinants of antibody binding, shedding light on the virus’s evolutionary strategies to evade immune responses and offering guidance for the design of therapeutics and vaccines. The analysis highlights that different antibodies employ distinct binding mechanisms, targeting unique epitopes on the RBD with varying degrees of conservation and flexibility. The comparative analysis of the binding mechanisms and resistance profiles of S309, S304, CYFN1006, and VIR-7229 reveals distinct molecular strategies employed by these antibodies to target the RBD of the SARS-CoV-2 S protein. These strategies can be broadly categorized into two paradigms: (1) conservation-driven binding, where antibodies exploit highly conserved residues critical for viral function, and (2) adaptability-driven binding, where antibodies utilize structural flexibility and compensatory interactions to tolerate mutations while maintaining neutralization efficacy. This dichotomy underscores the evolutionary trade-offs between targeting structurally constrained epitopes and accommodating antigenic drift through dynamic binding interfaces. S309 exemplifies a conservation-driven mechanism, targeting an epitope centered around T345, P337, R346, and L441—residues that are evolutionarily conserved across SARS-CoV-2 variants and related sarbecoviruses. In contrast, S304 adopts a more localized strategy, focusing its binding energy on fewer but highly sensitive residues, particularly T385 and K386. These residues serve as dominant energetic hotspots, forming a dense network of hydrogen bonds, electrostatic interactions, and hydrophobic packing that anchor the antibody to the inner face of the RBD β-sheet. While this focused interaction enables high-affinity binding, it also makes S304 particularly susceptible to escape mutations at these sites. CYFN1006 represents a hybrid approach, combining elements of both conservation-driven and adaptability-driven binding. Its epitope spans 17 RBD residues, including T345, K440, and T346—highly conserved positions that serve as critical anchors for binding. Unlike S309, however, CYFN1006 exhibits reduced reliance on K356, allowing it to maintain efficacy against variants carrying the K356T mutation, such as JN.1, KP.2, and KP.3. This broader epitope coverage distributes the energetic burden across multiple residues, enhancing resilience to mutations while preserving strong binding affinity. The combination of conserved residue targeting and distributed energy contributions makes CYFN1006 a prime example of a broad-spectrum therapeutic candidate capable of countering immune evasion. VIR-7229 distinguishes itself through an adaptability-driven mechanism, leveraging structural flexibility and backbone-mediated interactions to accommodate mutations within its epitope. Targeting a large and structurally complex region encompassing R403, F456, Y473, and L455, VIR-7229 forms extensive hydrophobic clusters and backbone hydrogen bonds that confer tolerance to substitutions such as F456L and L455S. This adaptability is particularly evident in its ability to neutralize Omicron subvariants KP.2 and KP.3, which carry multiple immune-evasive mutations. A recurring theme in the results is the role of highly conserved residues in stabilizing antibody–RBD interactions. For example, T345 and P337 are critical for S309 binding, with substitutions at these positions significantly impairing affinity. Similarly, T385 and K386 are indispensable for S304 binding. N343, A344, and T345 anchor CYFN1006 binding, while F456 and Y473 play central roles in VIR-7229 binding. The mutational scanning and MM-GBSA analyses reveal consistent patterns of immune evasion across variants, highlighting specific mutations that confer resistance to antibodies. A comparative analysis of the conservation status and mutation frequency of key epitope residues targeted by the four broadly neutralizing antibodies highlights the evolutionary constraints acting on each epitope (Supporting Information, Table S11). We also summarized key observations and findings by comparing differences in epitope location, dynamics, binding energetics mechanisms, localization of binding hotspots, and resistance profiles (Supporting Information, Table S12).

Taken together, these findings illustrate the diverse molecular strategies employed by neutralizing antibodies to engage the SARS-CoV-2 RBD. Conservation-driven antibodies like S309 and CYFN1006 prioritize stability through interactions with structurally constrained residues, offering broad protection against variants but facing challenges from convergent mutations. Adaptability-driven antibodies like VIR-7229 exploit structural flexibility and compensatory interactions to tolerate mutations, albeit with potential vulnerabilities at specific hotspot positions. S304 occupies a middle ground, relying on localized, high-energy interactions that provide potency but increase susceptibility to escape mutations.

## 5. Conclusions

This study provides critical insights into the molecular mechanisms governing the interactions between the SARS-CoV-2 spike RBD and neutralizing antibodies, including S309, S304, CYFN1006, and VIR-7229. By integrating advanced computational techniques such as molecular dynamics simulations, mutational scanning, and binding free energy calculations, we uncovered the structural, energetic, and evolutionary determinants of antibody–RBD binding. These findings reveal the intricate balance between viral immune evasion and the host immune response, offering a foundation for designing effective interventions against SARS-CoV-2 and its variants. The analysis highlights the diversity of binding mechanisms employed by different antibodies, each targeting unique epitopes with varying degrees of conservation and flexibility. Antibodies like S309 and CYFN1006 demonstrate broad-spectrum efficacy by leveraging highly conserved residues, making them less susceptible to mutations that disrupt binding. In contrast, antibodies such as S304 exhibit localized dependence on fewer residues, rendering them more vulnerable to escape mutations. VIR-7229 stands out for its adaptability to mutations, maintaining strong binding affinity even in the presence of key substitutions like F456L and L455S. A recurring theme in the results is the critical role of conserved residues in stabilizing antibody–RBD interactions. Residues such as T345, F456, and Y473 emerge as evolutionary “weak spots,” where mutations could destabilize the RBD structure or impair essential functions like ACE2 binding. These residues represent stable targets for therapeutic intervention, as they are less prone to random mutations due to their structural and functional importance. Understanding the evolutionary constraints on these residues can help predict future mutational trends and guide the design of vaccines and therapeutics that remain effective against emerging variants. The study also underscores the virus’s remarkable ability to evolve resistance through specific mutations, such as R346T and F456L, which consistently reduce antibody efficacy across multiple variants. These mutations reflect the selective pressure on the virus to evade immune responses while maintaining fitness. Broad-spectrum antibodies like CYFN1006 and VIR-7229, which tolerate such mutations, provide resilience to epitope variability and offer a high barrier to resistance, making them valuable tools in combating the rapidly evolving landscape of SARS-CoV-2. The findings emphasize the importance of targeting diverse epitopes and conserved residues to counteract viral evolution. By leveraging the principles of epitope conservation, structural adaptability, and energetic synergy, future antibody therapies with complementary binding mechanisms can be designed to stay ahead of the virus’s evolutionary trajectory, ensuring long-term protection against emerging variants.

## Data Availability

The original contributions presented in this study are included in the article/Supplementary Materials. Crystal structures were obtained and downloaded from the Protein Data Bank (http://www.rcsb.org, accessed on 20 February 2025). All simulations were performed using the NAMD 2.13 package obtained from website https://www.ks.uiuc.edu/Development/Download/, accessed on 1 March 2025. All simulations were performed using the all-atom additive CHARMM36 protein force field that can be obtained from http://mackerell.umaryland.edu/charmm_ff.shtml, accessed on 2 March 2025. The rendering of protein structures was done with the interactive visualization program UCSF ChimeraX package (https://www.rbvi.ucsf.edu/chimerax/, accessed on 15 February 2025) and Pymol (https://pymol.org/2/, accessed on 11 February 2025). All mutational heatmaps were produced using the developed software that is freely available at https://alshahrani.shinyapps.io/HeatMapViewerApp/, accessed on 16 March 2025.

## Supplementary Materials

The following supporting information can be downloaded at: https://www.mdpi.com/article/10.3390/v17060741/s1. Figure S1 presents structural details of the binding interfaces for SARS-CoV-2-RBD complexes with antibodies S309, S304, CYFN-1006.1/CYFN-1006.2, and VIR-7229. Figure S2 presents structural organization of the SARS-CoV-2-RBD complexes with CYFN-1006.1, CYFN-1006.2, and VIR-7229 antibodies. Datasets S1–S4 present the results of mutational scanning for different S309-RBD complexes. Datasets S5–S8 present the results of mutational scanning for different S304-RBD complexes. Datasets S9 and S10 present the results of mutational scanning for RBD complexes with CYFN-100.1 and CYFN-1006.2 antibodies. Datasets S11 and S12 present the results of mutational scanning for VIR-7229 binding with XBB.1.5 RBD and EG.5 RBD, respectively. Table S1 highlights the mutational landscape for Omicron variants explored in this study. Tables S2–S5 present the results of MM-GBSA computations for different S309-RBD complexes. Table S6 presents the results of MM-GBSA computations for S304-RBD complex. Tables S7 and S8 present the results of MM-GBSA computations for RBD complexes with CYFN-1006.1 and CYFN.1006-2, respectively. Tables S9 and S10 present the results of MM-GBSA computations for VIR-7229 complexes with XBB.1.5 RBD and EG.5 RBD. Table S11 presents a comparative analysis of the conservation status and mutation frequency of key epitope residues targeted by the four broadly neutralizing antibodies studied. Table S12 summarizes the major observations and highlights differences in the epitopes, dynamics, binding mechanisms, binding hotspots, and escape resistance profiles for the examined antibodies.
