# Supplementary material for: Integrative Computational Modeling of Distinct Binding Mechanisms for Broadly Neutralizing Antibodies Targeting SARS-CoV-2 Spike Omicron Variants: Balance of Evolutionary and Dynamic Adaptability in Shaping Molecular Determinants of Immune Escape
Source: Viruses. 2025 May 22;17(6):741. doi: 10.3390/v17060741 (PMC12197533; doi:10.3390/v17060741)
Supplement: Supplementary file 1 [file viruses-17-00741-s001.zip › SUPPLEMENTARY_MATERIALS_REVISION.pdf]

Supplementary Materials

Integrative Computational Modeling of Distinct Binding Mechanisms for Broadly Neutralizing Antibodies Targeting SARS-CoV-2 Spike Omicron Variants: Balance of Evolutionary and Dynamic Adaptability in Shaping Molecular Determinants of Immune Escape

Mohammed Alshahrani [1], Vedant Parikh [1], Brandon Foley [1], and Gennady Verkhivker [1,2*]

[1] Keck Center for Science and Engineering, Graduate Program in Computational and Data Sciences, Schmid College of Science and Technology, Chapman University, Orange, CA 92866, United States of America alshahrani@chapman.edu (M.A); vedpar31@gmail.com (V.P.); brfoley@chapaman.edu (B.F.); verkhivk@chapman.edu (G.V).

[2] Department of Biomedical and Pharmaceutical Sciences, Chapman University School of Pharmacy, Irvine, CA 92618, United States of America

* Correspondence: verkhivk@chapman.edu; Tel.: +1-714-516-4586 (G.V)

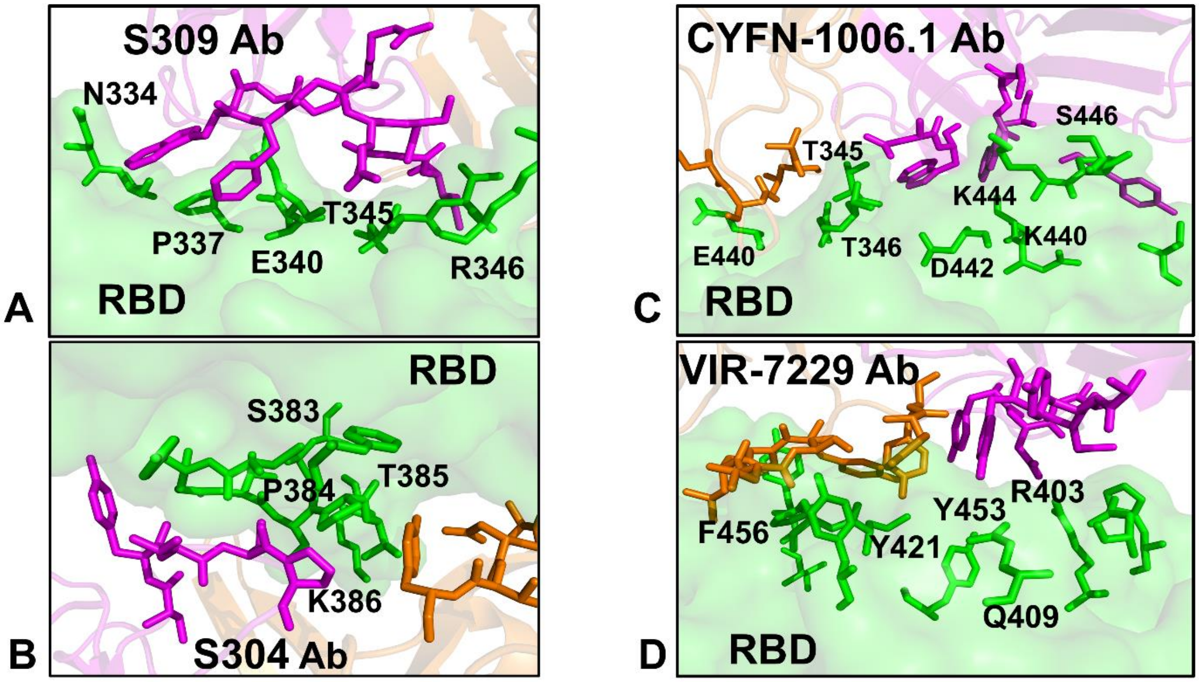

Figure S1. Structural details of the binding interfaces for SARS-CoV-2-RBD complexes with antibodies S309 (A), S304(B), CYFN-1006.1 and CYFN-1006.2 (C), and VIR-7229 (D). The S-RBD structure is shown in green transparent surface. The heavy chains of antibodies are shown in orange ribbons and light chains are in magenta-colored ribbons. The binding interface residues are shown in sticks and colored according to the chain they belong to : RBD residues are in green sticks and antibody residues are in orange and magenta sticks. The key RBD binding interface residues for each of the antibodies are annotated on panels (A)-(D).

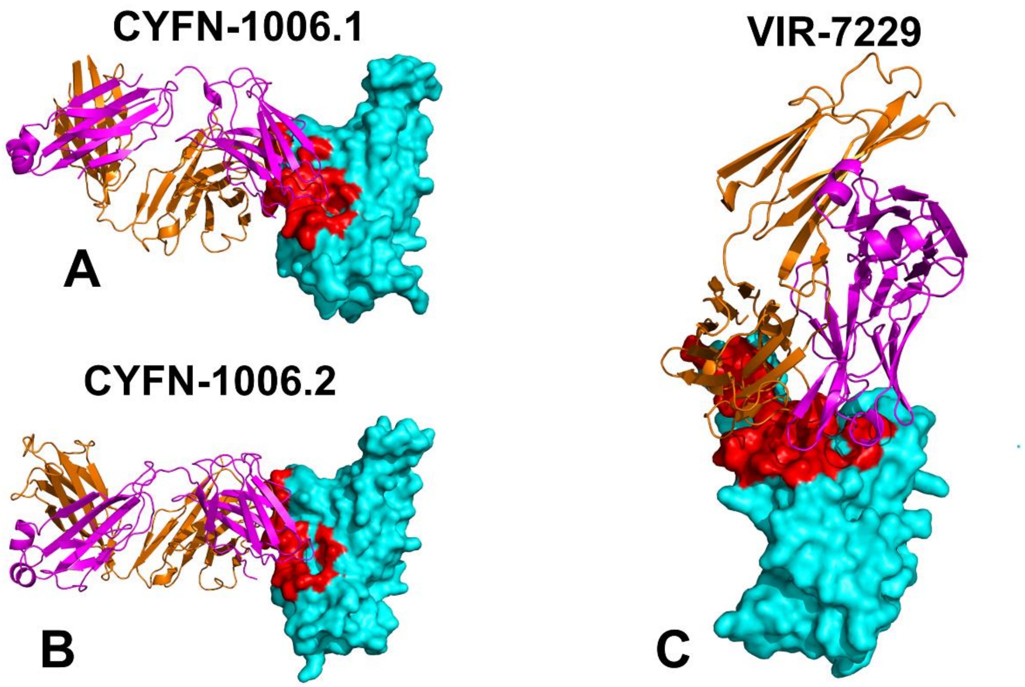

Figure S2. Structural organization of the SARS-CoV-2-RBD complexes with CYFN-1006.1 (A), CYFN-1006.2 (B), and VIR-7229 antibodies (C). The S-RBD structure is shown in cyan surface. The heavy chains of antibodies are shown in orange ribbons and light chains are in magenta-colored ribbons. The binding epitope residues are shown in red surface.

Table S1. Mutational landscape of the Omicron variants.

Omicron Variant	Mutational landscape
BA.1	A67, T95I, G339D, S371L, S373P, S375F, K417N, N440K, G446S, S477N, T478K, E484A, Q493R, G496S, Q498R, N501Y, Y505H, T547K, D614G, H655Y, N679K, P681H, N764K, D796Y, N856K, Q954H, N969K, L981F
BA.2	T19I, G142D, V213G, G339D, S371F, S373P, S375F, T376A, D405N, R408S, K417N, N440K, S477N, T478K, E484A, Q493R, Q498R, N501Y, Y505H, D614G, H655Y, N679K, P681H, N764K, D796Y, Q954H, N969K
XBB.1.5	T19I, V83A, G142D, Del144, H146Q, Q183E, V213E, G252V, G339H, R346T, L368I, S371F, S373P, S375F, T376A, D405N, R408S, K417N, N440K, V445P, G446S,N460K, S477N, T478K, E484A, F486P, F490S, R493Q reversal, Q498R, N501Y, Y505H, D614G, H655Y, N679K, P681H, N764K, D796Y, Q954H, N969K
BA.2.86	T19I, R21T, S50L, del69-70,V127F, delY144, F157S, R158G, delN211, L213I, L226F, H25N,A264D, I332V, D339H, K356T, R403K, V445H, G446, N450D, L452W, N460K, N481K, del V483, A484K, F486P, R493Q, E554K, A570V, P612S, I670V, H68R, D939F, P1143L
JN.1	T19I, R21T, S50L, del69-70,V127F, delY144, F157S, R158G, delN211, L213I, L226F, H25N,A264D, I332V, D339H, K356T, R403K, V445H, G446, N450D, L452W, **L455S,** N460K, N481K, del V483, A484K, F486P, R493Q, E554K, A570V, P612S, I670V, H68R, D939F, P1143L
KP.2	T19I, R21T, S50L, del69-70,V127F, delY144, F157S, R158G, delN211, L213I, L226F, H25N,A264D, I332V, D339H, **R346T**, K356T, R403K, V445H, G446, N450D, L452W, **L455S, F456L**, N460K, N481K, del V483, A484K, F486P, R493Q, E554K, A570V, P612S, I670V, H68R, D939F, **V1104L,** P1143L
KP.3	T19I, R21T, S50L, del69-70,V127F, delY144, F157S, R158G, delN211, L213I, L226F, H25N,A264D, I332V, D339H, K356T, R403K, V445H, G446, N450D, L452W, **L455S, F456L**, N460K, N481K, del V483, A484K, F486P, **Q493E**, E554K, A570V, P612S, I670V, H68R, D939F, **V1104L,** P1143L

Table S11. A comparative analysis of the conservation status and mutation frequency of key epitope residues targeted by S309, S309, CYFN-1006 and VIR-7229 antibodies.

Antibody	Targeted RBD residues	Conservation level	Mutational frequency in circulating variant	Escape mutations
**S309**	T345, R346, P337, L441, N343, K356, A344, K444	**T345, N343, A344, L441:** highly conserved **R346, P337, K356, K444:** variable	**R346T/S/K:** common in CH.1.1, CA.3.1, JN.1, KP.2, KP.3 **P337L:** present in BQ.1.1, XBB.1.5 **K356T:** prevalent in JN.1, KP.2, KP.3	High sensitivity to **R346T** and **K356T** , leading to reduced binding and neutralization in later Omicron subvariants.
**S304**	T385, K386, Y380, P384, S383, F377, C379, V382	**T385, K386, P384:** moderately conserved but functionally critical **Y380, S383, F377:** variable	**T385K/D/E/R:** frequent in immune-evasive lineages **K386D/R:** emerging in B.1.91 and other VOCs **Y380Q:** found in B.1.91 lineage	Strong correlation between mutations at **T385** and **K386** and loss of binding; local dependence makes it vulnerable to escape.
**CYFN-1006**	T345, K440, T346, N343, A344, L441, P445, P499	**T345, N343, A344, L441:** highly conserved **T346, K440, P445, P499:** moderately conserved	**T345A/N/V:** rare **T346I/N/S:** moderate frequency **K356T:** tolerated due to low involvement in binding	Broad neutralization maintained due to targeting **conserved core residues** ; less affected by mutations like **K356T** , making it effective against JN.1, KP.2, and KP.3.
**VIR-7229**	R403, F456/L456, Y473, Y489, L455, R457, K458	**F456/L456, Y473, Y489:** hydrophobic cluster with high conservation **R403, L455, R457, K458:** variable	**F456L:** tolerated via compensatory interactions **L455S:** tolerated in KP.3 **F486P/K/E:** strongly disruptive, associated with escape	Structural adaptability allows tolerance of **F456L** and **L455S** , but **F486P/K/E** mutations severely impair binding; high barrier to resistance.
